# Decolonising research, advocacy and public policy on healthy diets

**DOI:** 10.1371/journal.pgph.0002140

**Published:** 2023-07-24

**Authors:** Mélissa Mialon

**Affiliations:** Trinity College Dublin, Dublin, Ireland; PLOS: Public Library of Science, UNITED STATES

Large, global commercial actors fuel every region of our world with ultra-processed products and other unhealthy commodities [1]. Ultra-processed products are extremely profitable for food companies and are aggressively marketed to children, women, Black, Indigenous, People of Color (BIPOC), amongst other groups [1]. With markets saturated in high-income countries, food companies also penetrate low- and middle-income countries [1]. Yet, there is ample evidence that the consumption of ultra-processed products leads to ill-health and deepens inequities [2]. Ultra-processed and other unhealthy products therefore have a disproportionate effect on poor people and other groups targeted by the food industry.

These groups already suffer from other effects of coloniality and food industry practices continue the legacy of coloniality. There are discussions about the history of some large commercial actors in the modern food industry, such as the sugar industry, and its legacy from the Dutch and British trading companies [3]. Today, corporations not only target BIPOC and other communities with unhealthy products, they also use economic and other crises, like COVID-19, to promote a good image for themselves with their "corporate social responsibility" (CSR) and other philanthropic activities [4]. This is particularly seen as important in low income settings, which have limited capacity to protect themselves from crises. Large food companies in Brazil, for example, donated their products (including ultra-processed ones) to food banks during the COVID-19 pandemic [5]. CSR and philanthropy help commercial actors secure a favourable public opinion and build relationships with decision-makers and other third parties and perpetuates that image of food industry actors as saviours, who come with funding and knowledge on how to address health issues. However, framing CSR and philanthropy as key solutions ignores the central role of the food industry in fueling communities with unhealthy products and thus furthering health inequities. Countries where global, large corporations are headquartered, such as the U.S. and European nations, even serve as proxies for that corporate influence. This was evidenced a few years ago when the U.S.A., home to some of the largest baby food companies, threatened to impose trade sanctions on Ecuador if the Latin American country supported a UN resolution on breastfeeding [6]. Corporations and high-income countries, in fact, perpetuate a colonial relationship with low- and middle-income countries.

That legacy of coloniality has also permeated research, advocacy and public policy on healthy diets, which continue to be dominated by powerful commercial actors. Among the top 10 nutrition journals, 13% of publications have involvement of the food industry, through authorship and funding [7]. This is problematic, as the industry’s involvement in research leads to biased research on healthy diets, and advocacy and public policy based on that science. For example, research where a conflict of interest or funding from the food industry was declared is five times more likely to report no association between the consumption of sugar-sweetened beverages and weight gain, compared to independent research [8].

Moreover, corporations have a central role in international fora: the United Nations (UN) Global Compact is based on voluntary commitments made by corporations [9], UN Sustainable Development Goal 17 focuses on partnerships with commercial actors, and there are numerous multi-stakeholder mechanisms where commercial actors have a key role in decision-making, for example through the Scaling Up Nutrition (SUN) initiative—a global public-private partnership [10]. The UN Food Systems Summit in 2021 was perhaps the epitome of all of this, with corporations being given a prominent space in the Summit programme, with little transparency about governance and other ethical issues [11].

Confronted with that corporate capture, groups of academics, advocates, and policymakers are working towards a human-rights-based approach to healthy diets. Some of these groups include international coalitions such as La Via Campesina [12], and country-specific alliances such as the Aliança pela Alimentação Adequada e Saudável (Alliance for Adequate and Healthy Food) in Brazil [13]. Those groups promote the local production of minimally processed foods, BIPOC (Black, Indigenous, and people of colour) and peasants’ knowledge and rights, and food sovereignty, amongst other principles for healthy diets. Some of these groups argue that commercial actors that produce unhealthy commodities should not influence research, advocacy, and public policy, given their conflicting financial interests.

Recent academic work argues that there could be a consensus on what interactions might be acceptable between public health researchers and the food industry [14], but these solutions ignore the power asymmetries between those different actors, and their relationships with coloniality. There is an urgent need to better understand how some of the legacies of coloniality, including imperialism, patriarchy, capitalism, elitism, and privatization, impact research, advocacy and public policy on healthy diets. These legacies are the causes of suffering for many and have a direct impact on population health [15]. There must be more space for critical thinking in dietetics and health training. We need to think about the role of international versus local researchers in global nutrition research conducted in low-income countries, and amplify local knowledge and local authorship in research. Researchers, advocates and policymakers in low and middle income countries—as well as in the diaspora—have immense collective knowledge. We need national health strategies that explicitly include the protection of public policy from commercial vested interests, and rather give a central space to marginalized voices.

The commercial capture of research, advocacy and public policy on health diets is a direct product of coloniality, and it is urgent to address its root causes if we are to protect population health.
